# Transient Receptor Potential Vanilloid-1 (TRPV1) Alleviates Hepatic Fibrosis via TGF-*β* Signaling

**DOI:** 10.1155/2022/3100943

**Published:** 2022-07-21

**Authors:** Ke Qian, Xiaohua Lei, Guoxing Liu, Yu Fang, Chengzhi Xie, Xiaolong Wu, Qiang Liu, Gao Liu, Zhenyu Cao, Ju Zhang, Tao Kuang, Likun Yan, Jie Fu, Huihui Du, Zhiqiang Liu, Yuan Chu, Ge Xu, Hirofumi Yamamoto, Masaki Mori, Xin M. Liang, Xundi Xu

**Affiliations:** ^1^Division of Hepato-Biliary-Pancreatic Surgery, Department of Surgery, The Second Xiangya Hospital, Central South University, Hunan Provincial Key Laboratory of Hepatobiliary Disease Research, Changsha, Hunan 410011, China; ^2^The First Affiliated Hospital, Department of Hepato-Biliary-Pancreatic Surgery, Hengyang Medical School, University of South China, Hengyang, Hunan, China; ^3^Department of Molecular Pathology, Division of Health Sciences, Graduate School of Medicine, Osaka University, 1-7 Yamadaoka, Suita, Osaka 565-0871, Japan; ^4^Department of Surgery and Science, Graduate School of Medical Sciences, Kyushu University, Fukuoka 812-8582, Japan; ^5^Wellman Center for Photomedicine, Department of Medicine, Division of Endocrinology, Massachusetts General Hospital, VA Boston Healthcare System, Beth Israel Deaconess Medical Center, Harvard Medical School, Boston, Massachusetts 02115, USA; ^6^Department of general surgery, Southern China Hospital, Health Science Center, Shenzhen University, No. 1, Fuxin Road, Pinghu Street, Longgang District, Shenzhen, China

## Abstract

Hepatic fibrosis is a major global health problem and considered a leading cause of liver-related morbidity and mortality worldwide. Although previous studies have suggested that transient receptor potential vanilloid-1 (TRPV1) is protective against cardiac and renal fibrosis, its functional role in hepatic fibrosis has remained elusive. Herein, we characterize the effects of TRPV1 on carbon tetrachloride- (CCl_4_-) induced mice, *in vitro* transforming growth factor-*β*- (TGF-*β*-) treated hepatic stellate cells (HSCs), and human fibrosis specimens. Finally, our results demonstrated the significant TRPV1 downregulation in human liver fibrosis tissues. Knocking out TRPV1 significantly increased the expression of various hepatic fibrosis markers, while the expression of these biomarkers declined markedly in capsaicin-activated mice. Moreover, our study revealed that knocking down TRPV1 would enhance the promotive effect of TGF-*β* on HSC proliferation, cell cycle, cell apoptosis, and ECM expression. Also, such promotive effect can be partially reversible by capsaicin, an exogenous activator of TRPV1. Collectively, the obtained data suggest that TRPV1 may alleviate CCl_4_-induced hepatic fibrosis and attenuate the effect of TGF-*β* on HSC activation, proliferation, and apoptosis, which overall implies that targeting TRPV1 channel activity may be an effective therapeutic strategy for treating hepatic fibrosis.

## 1. Introduction

Hepatic fibrosis, a significant global health risk with high morbidity and mortality rates [1], develops as a result of a dynamic ubiquitous response to most chronic liver injuries of any etiologies, including viral infection, alcohol abuse, and nonalcoholic fatty liver diseases (NAFLDs) [2–4]. Due to impaired regenerative capability, the initially beneficial wound healing process of the liver becomes pathogenic resulting in characteristic dysregulated excessive extracellular matrix (ECM) production and deposition, aberrant hepatocyte (scar tissue) formation, distortion of liver vasculature and architecture, portal hypertension, and eventually organ failure. [5–7] Although emerging evidence has suggested that portal fibroblasts [5, 8, 9] and myofibroblasts of bone marrow origin [10] may also be significant collagen-producing cells in the chronically injured liver, activation of perisinusoidal resident hepatic stellate cells (HSCs) into proliferative, contractile, and fibrogenic myofibroblast-like phenotypes remains the fundamental theme for driving the hepatic fibrosis response and the development of cirrhosis [11–15]. Under physiological conditions, quiescent HSCs (qHSCs) are the major vitamin A-storing cells and typically reside in the perisinusoidal space of Disse, a recess between endothelial cells of sinusoids and hepatocytes, with the expression of distinctive markers, such as glial fibrillary acid protein (GFAP), synaptophysin, nerve growth factor p75 (NGFR1), and lecithin retinol acyltransferase (Lrat) [5, 16, 17]. Following liver injury, qHSCs are activated by fibrogenic cytokines, such as transforming growth factor-*β* (TGF-*β*), angiotensin II, and leptin, resulting in the downregulation of vitamin A expression in lipid droplets, the migration towards pericentral areas, and the transdifferentiation into *α*-smooth muscle actin (*α*-SMA) and type I collagen- (COL1A1-) upregulated fibrogenic myofibroblast-like phenotypes [5, 16, 18, 19].

Transient receptor potential (TRP) channels are polymodal receptors that can be activated by a wide variety of physical and chemical stimuli to facilitate sensory transduction [20, 21]. The TRP superfamily consists of 28 different genes and is further subcategorized into 7 different subfamilies (TRPA, TRPC, TRPM, TRPML, TRPN, TRPP, and TRPV) with nonselective variable cation permeability [22]. The capsaicin receptor, TRPV1, is a nociceptor at the plasma membrane whose activation leads to higher Ca^2+^ influx through a highly permeable cation channel and evocation of pain [23]. Compelling evidence has suggested that TRPV1 is not only essential for the development of heat hyperalgesia in response to tissue inflammation [24] but also vital in triggering various stress-related cellular responses [25]. Furthermore, continued studies have also suggested that TRPV1 inhibition may suppress inflammation and fibrosis/scarring in various pathological conditions, including toxic lung injuries, inflammation during the development of asthma and chronic obstructive pulmonary disease (COPD), cardiac and intestinal renal fibrosis, atherosclerosis, congestive heart failure, pulmonary and systemic hypertension, hemorrhagic shock, vascular remodeling, and even certain types of cancer, thereby eliciting improved wound healing outcomes [26–33].

In this study, the functional significance of TRPV1 in HSC activation and hepatic fibrosis progression was evaluated based on histopathology, serology, cellular, and molecular characteristics using microdissected specimens obtained from either patients with a similar stage of cirrhosis or classic carbon tetrachloride- (CCl_4_-) induced murine fibrosis model, namely, CCl_4_-induced WT and KO mice underwent various experimental procedures. Levels of *α*-SMA and COL1A1 expressions and representative markers of antioxidant metabolism were used to evaluate the effect of TRPV1 on HSC activation and proliferation upon TGF-*β* stimulation. To further elucidate the protective effect of TRPV1 against fibrogenesis, capsaicin was utilized to rescue TGF-*β*-activated HSCs in terms of extracellular matrix (ECM) production and cell proliferation. Taken together, our findings suggested a pathological role of TRPV1 in the development of CCl_4_-induced hepatic fibrosis, as well as the activation and proliferation of HSC, implying that TRPV1 might be a promising therapeutic target for developing antifibrosis strategies with improved outcomes.

## 2. Materials and Methods

### 2.1. Laboratory Animal

*TRPV1^−/−^* mice (C57BL/6J, *TRPV1^−/−^*) and WT mice (C57BL/6J) were obtained from the Model Animal Research Center of Nanjing University (Jiangsu, China). Homozygous *TRPV1^−/−^* mice were inspected, quarantined, sequencing identified, and then grouped for future experiments.

### 2.2. Reagents

CCl_4_ was obtained from the Shantou Xilong Chemistry Plant (Shantou, Guangdong, China). Dimethylsulfoxide (DMSO) was purchased from Sigma-Aldrich. (St. Louis, MO, USA). Antibodies against TRPV1 (ab10296), *α*-SMA (ab5694), COL1A1 (ab34710), and *β*-actin (ab8226) were obtained from Abcam (Cambridge, MA, USA), same as the picro-sirius red staining kit (ab150681). TGF-*β* was purchased from R&D Systems (Minneapolis, MN, USA). Secondary antibodies for goat anti-rabbit immunoglobulin (IgG), horse radish peroxidase (HRP), and goat anti-mouse IgG HRP were purchased from Santa Cruz Biotechnology (Santa Cruz, CA, USA). Capsaicin was purchased from Sigma (Santa Clara, CA, USA).

### 2.3. CCl_4_-Induced Hepatic Fibrosis Mouse Model

Hepatic fibrosis was induced by an 8-week treatment for both adult *TRPV1^−/−^* and WT mice with 1 *μ*l/g body weight of CCl_4_/olive oil (1 : 9 vol/vol) via intraperitoneal injection (twice per week) as described [34]. *TRPV1^−/−^* or WT mice in the control group were treated intraperitoneally twice per week with 1 *μ*l/g body weight of olive oil. 24 h after the final CCl_4_ injection, serum was isolated for analyzing the serum levels of ALT and AST; mice were then sacrificed and liver tissues (*n* > 5) were harvested for further analysis. For capsaicin reversal experiments, WT mice had first undergone six weeks of CCl_4_ treatment via intraperitoneal injection (twice per week), followed by daily intraperitoneal injection (once per day) of either saline or 2 mg/kg capsaicin. 24 h after the final CCl_4_ injection, mice were sacrificed and liver tissues (*n* > 5) were harvested for further analysis. All animal experiment-related protocols were approved by the University Animal Care and Use Committee of Central South University.

### 2.4. Cell Culture, Cell Transfection, and TGF-*β* Treatment

Human hepatic stellate cells (HSCs) were received from Procell Life Science & Technology (Wuhan, China). HSCs were cultured in Dulbecco's modified Eagle's medium (DMEM) (Sigma-Aldrich, St. Louis, MO, USA) supplemented with 100 U/ml penicillin and 100 mg/ml streptomycin in a humidified incubator at 37°C with 5% CO_2_. To achieve the TRPV1 knockdown status, HSCs were cultured in serum-free DMEM for 12 h and then subjected to transfection with si-TRPV1 (Shanghai Sangon Biological and Technological, Shanghai, China) according to the manufacturer's instruction. Oligonucleotide was transfected into HSCs by Lipofectamine 2000 (Invitrogen, Carlsbad, CA, USA), following the protocol recommended by the manufacturer. Cells were collected for further analysis after 48 h of transfection. Prior to any TGF-*β*-related experiments, HSCs were treated with 5 ng/ml of TGF-*β* for 24, 48, or 72 h.

### 2.5. Histopathology

Mouse liver tissues were fixed in 10% paraformaldehyde, embedded in paraffin wax, and thin sectioned (4 *μ*m thickness) before staining. To quantify the hepatic fibrosis area, the prepared sections were stained with picro-sirius red and observed using an Olympus Provis microscope equipped with a CCD camera (Tokyo, Japan), as described previously [35, 36]. For Oil Red O staining, liver sections were stained with 0.3% Oil Red O in triethyl phosphate in water (60% *v*/*v*) for 30 min [37]. The sections were then observed using a BX-60 microscope with a DP-11 digital camera (Olympus, GmbH, Hamburg, Germany). To ensure data accuracy and reliability, a minimum of 5 chicks was selected for analysis per group, at least 5 sections were studied per chick, and at least 7 randomly selected regions of interest (ROIs) per section were analyzed.

### 2.6. Serum Biochemical Parameters

Upon completion of the final CCl_4_ injection, serum was collected by orbital venous plexus blood collection. The mouse blood serum was separated, the levels of AST and ALT were evaluated using the standard enzymatic assay kits, and the serum levels of hyaluronic acid, laminin, collagen type IV, and procollagen III were measured using the radioimmunoassay kits (Haiyan Medical Biotechnology Center, Shanghai, China). Each assay is a colorimetric assay with detection of a highly colored end product measured at 490–520 nm by a spectrophotometer (Hitachi 736-10, Hitachi, Japan). The absorbance of each end product is proportional to the enzyme's activity.

### 2.7. Experiment on Antioxidant Metabolism

Superoxide dismutase (SOD) (A001-1) and total antioxidant capacity (T-AOC) (A015) were obtained from Nanjing Institute of Biological Engineering. The SOD and T-AOC activities were determined via xanthine oxidation (XO) and chemical colorimetry, respectively.

### 2.8. MTT Assay

Following transfection, cell proliferation was evaluated using MTT assay (Sigma-Aldrich, MO, USA), per manufacturer's instructions. In brief, cells were seeded in 96-well plates at a density of 5 × 10^3^ cells per well and cultured with siRNA-TRPV1 at 37°C in a humid chamber with 5% CO_2_ for 48 h. 24 h after transfection or capsaicin treatment with or without TGF-*β*, 20 *μ*l of 5 mg/ml MTT (dimethyl thiazolyl diphenyl tetrazolium, Sigma-Aldrich, St. Louis, MO, USA) was then added into each well and incubated with cells at 37°C for 4 h. The supernatant was then discarded, following a 200 *μ*l of DMSO addition to each well to dissolve the formazan. The optical density (OD) was measured at 490 nm. The percentage of viability was calculated using the following equation: viability percentage = *T*/*C* × 100%, where *T* is the absorbance of the transfection group and *C* the absorbance of the control group.

### 2.9. BrdU Incorporation Assay

BrdU assays were performed to determine DNA synthesis at 24 and 48 h after transfecting HSCs with si-TRPV1 or treating with capsaicin in the presence or absence of TGF-*β*. HSCs were cultured for either 24 or 48 h and incubated with a final concentration of 10 *μ*M BrdU (BD Pharmingen, San Diego, CA, USA) for 2 to 24 h. At the end of the incubation period, the medium was removed; the cells were fixed for 30 min at RT, incubated with peroxidase-coupled anti-BrdU-antibody (Sigma-Aldrich, St. Louis, MO, USA) for 60 min at RT, washed with PBS, and incubated with peroxidase substrate (tetramethyl benzidine) for 30 min; and the absorbance values were measured at 450 nm.

### 2.10. Flow Cytometry

For cell cycle analysis, cells were washed twice in ice-cold PBS, resuspended in 1 ml ice-cold 70% ethanol with gentle vortexing, and incubated overnight at 4°C. Cells were then washed with 1x PBS and resuspended in buffer containing 10 *μ*g/ml propidium iodide, 10 *μ*g/ml RNase A, and 0.1% Triton X-100 and incubated for 30 min at room temperature in the dark. After incubation, the DNA content in the cells was measured by a Guava Easy-Cyte 8HT flow cytometer (Merck Millipore, Darmstadt, Germany) excited with blue laser (488 nm) and analyzed using InCyte software.

### 2.11. Western Blot Analysis

Mouse liver tissues or cultured cells were lysed using RIPA buffer (Sigma-Aldrich, USA), complemented with Complete Protease Inhibitor Cocktail (Roche, USA). The lysates were transferred to 1.5 ml tube and kept at −20°C before use. SDS-PAGE was conducted to separate the cellular proteins. And all the cellular proteins within this study were separated by 5% stacking gel and 10% running gel. The molecular weight of candidate proteins was referred to the information of the prestained SeeBlue rainbow marker (Invitrogen, USA) loaded in parallel. The membranes were probed with the following antibodies: ab10296 (for TRPV1), ab5694 (for *α*-SMA), ab34710 (for COL1A1), and ab8226 (for *β*-actin). Following incubation with primary antibodies, blots were washed for four times in TBS/Tween-20 before the 1 h incubation in goat anti-rabbit horseradish peroxidase conjugate antibody at 1 : 10000 dilution in TBS/Tween-20 containing 5% skim milk. After washing extensively in TBS/Tween-20, the blots were then processed with distilled water for detection of antigen using the enhanced chemiluminescence system. The blots were visualized with the ECL-chemiluminescent kit (ECL-plus, Thermo Scientific) and detected on a Fujifilm developer (Fujifilm, Japan).

### 2.12. Immunofluorescent (IF) Staining

After being treated with TGF-*β* (5 ng/ml) for 24 h, HSCs grown on the coverslips were fixed in 4% paraformaldehyde and stained with 1 : 100 ab5694 (anti-*α*-SMA) and 1 : 500 ab34710 (anti-COL1A1) antibodies, followed by fluorescein isothiocyanate (FITC) and tetramethyl rhodamine iso-thiocyanate conjugated secondary antibodies using standard procedures. Cell nuclei were stained with 4′,6-diamidino-2-phenylindole (DAPI, ab104139, Abcam, USA), followed by confocal microscopy (LSM 510, Zeiss, Germany).

### 2.13. Human Subject and Immunohistochemistry

40 randomly selected paraffin-embedded liver fibrosis tissues from end-stage cirrhosis patients who received a liver transplantation and 10 randomly selected paraffin-embedded nonneoplastic tissues as normal control from liver hemangioma patients who received hepatectomy at the Department of Surgery, The Second Xiangya Hospital, Central South University, from January 2010 to December 2019, were used. The sections were incubated with TRPV1 antibodies (1 : 200, GTX54762, GeneTex, CA, USA) via a streptavidin peroxidase-conjugated method as described previously [38]. TRPV1 expression was determined according to the intensity of immunohistochemistry staining: 0 (no), 1 (weak), 2 (moderate), and 3 (strong). The TRPV1 expression was also divided into a high-expression group [2–4] and a low-expression group (0–1) for further analysis. This protocol was approved by The Second Xiangya Hospital institutional Ethics Committee for human subject research.

### 2.14. Statistical Analyses

To ensure reliability and repeatability in the approach, three repetitions were performed and a minimum of five samples was analyzed per condition. All reported data are represented as the mean values of the corresponding conditions with standard deviation (SD) as the error bars to demonstrate the mean value variability. All statistical analyses for data were performed using SPSS 16.0 software (Chicago, IL, USA). Between two groups, data were analyzed using Student's *t*-test, while one-way ANOVA tests, followed up with multiple comparison tests, were utilized to evaluate the statistical significance among three or more groups. Statistical significance was defined as *p* < 0.05.

## 3. Results

### 3.1. TRPV1 Modulates CCl_4_-Induced Hepatic Fibrosis in Mice

**Figure 1:**
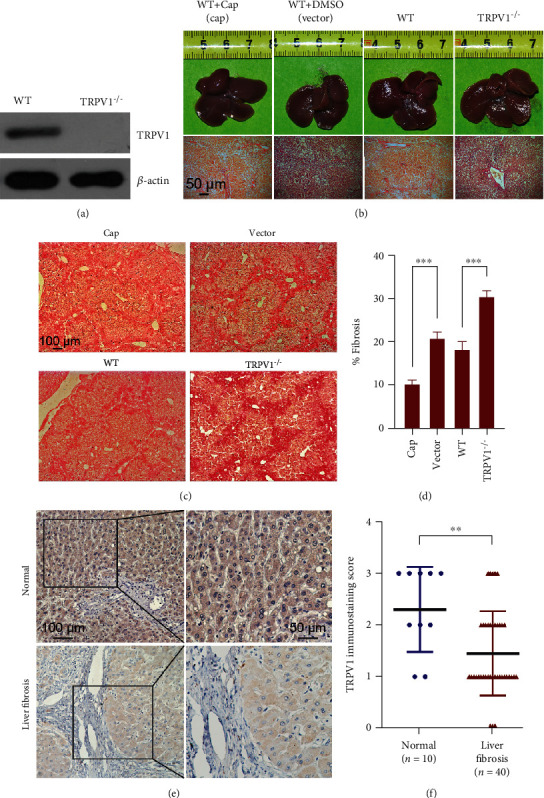
TRPV1 modulates CCl_4_-induced hepatic fibrosis in mice, and its expression is downregulated in human liver fibrosis. (a) TRPV1 protein expression in *TRPV1^−/−^* mice was measured through Western blot analysis. (b) Morphology of whole livers and H&E-stained liver sections (400x magnification) from the four CCl_4_-induced hepatic fibrosis models (WT with capsaicin intraperitoneal injection, WT with DMSO intraperitoneal injection, WT, *TRPV1^−/−^*, all under CCl4 treatment). (c) Pathological morphology of the four animal models was shown by picro-sirius red staining. (d) Quantitative analysis of the fibrosis area. Data are presented as mean ± SD (*n* = 5 mice). ^∗∗∗^*p* < 0.001. (e) Representative immunohistochemistry images of TRPV1 expression in normal control liver and liver fibrosis. (f) The semiquantitative levels of TRPV1 expression by immunohistochemistry in liver paraffin sections from 10 normal control and 40 liver fibrosis patients. ^∗∗^*p* < 0.001. Data are presented as mean ± SD.

To evaluate the function of TRPV1 in hepatic fibrosis, two murine hepatic fibrosis comparison models were utilized in this study, namely, the CCl_4_-treated TRPV1 KO vs. WT and WT receiving vehicle vs. WT receiving capsaicin after CCl_4_ stimulation. Before treating the mice with CCl_4_, Western blot analyses on TRPV1 protein levels from TRPV1^−/−^ mice tissues were performed to obtain the baseline measurement. As shown in Figure 1(a), TRPV1 was not detectable in the *TRPV1^−/−^* group. Standard intraperitoneal injection or 0.01% capsaicin intraperitoneal injection for WT mice was conducted after six weeks of CCl_4_ treatment. Subsequent examinations of macroscopic appearances of the livers have concluded that compared with the livers in the capsaicin intraperitoneal injection WT group with a regular and smooth surface, the livers in the other three groups, including WT mice with standard intraperitoneal injection and *TRPV1^−/−^* mice, were puffy, stiff, and acquired an irregular and granular surface; the CCl_4_-induced fibrosis appearances were more severe in *TRPV1^−/−^* mice (Figure 1(b)). Meanwhile, picro-sirius red staining was employed to quantify the hepatic fibrosis area.  Figure 1(c) shows a lobular architecture with central veins and radiating hepatic cords in the capsaicin intraperitoneal injection WT group; while CCl_4_ treatment resulted in damaged lobular architectures, severe vacuolar hepatocyte degeneration, enlarged fibrous septa, pseudolobule formations, and increased deposition of collagen fibers, similar to liver appearances, CCl_4_-treated *TRPV1^−/−^* mice had a higher fibrotic degree than their CCl_4_-treated WT counterparts and the area of fibrosis was significantly reduced by capsaicin while increased in *TRPV1^−/−^* mice (*p* < 0.001) as shown in Figure 1(d). Overall, the morphological analysis of the aforementioned four groups suggested fewer fibrosis per field in capsaicin-treated WT mice when comparing with vector-treated WT mice (*p* < 0.001) and significantly more fibrosis per field in *TRPV1^−/−^* mice when comparing with WT (*p* < 0.001).

### 3.2. TRPV1 Expression Is Downregulated in Human Liver Fibrosis

Immunohistochemical staining revealed that TRPV1 expression was significantly downregulated comparing human cirrhotic liver tissue specimens to healthy controls (*p* < 0.01, Figure 1(e)). High expression of TRPV1 was observed in 80% (8/10) of the normal liver tissues, while only 40% (16/40) of the fibrosis specimens demonstrated a similar level of TRPV1 expression. The cohort of 40 patients included hepatitis B-related cirrhosis, alcoholic cirrhosis, and autoimmune hepatitis-related cirrhosis. Immunohistochemical analysis also suggested a major reduction of hepatic TRPV1 expression in cirrhotic tissues compared with normal human liver tissues, regardless of etiology (data not shown).

### 3.3. Protective Effect of TRPV1 on the CCl_4_-Induced Mouse Model

**Figure 2:**
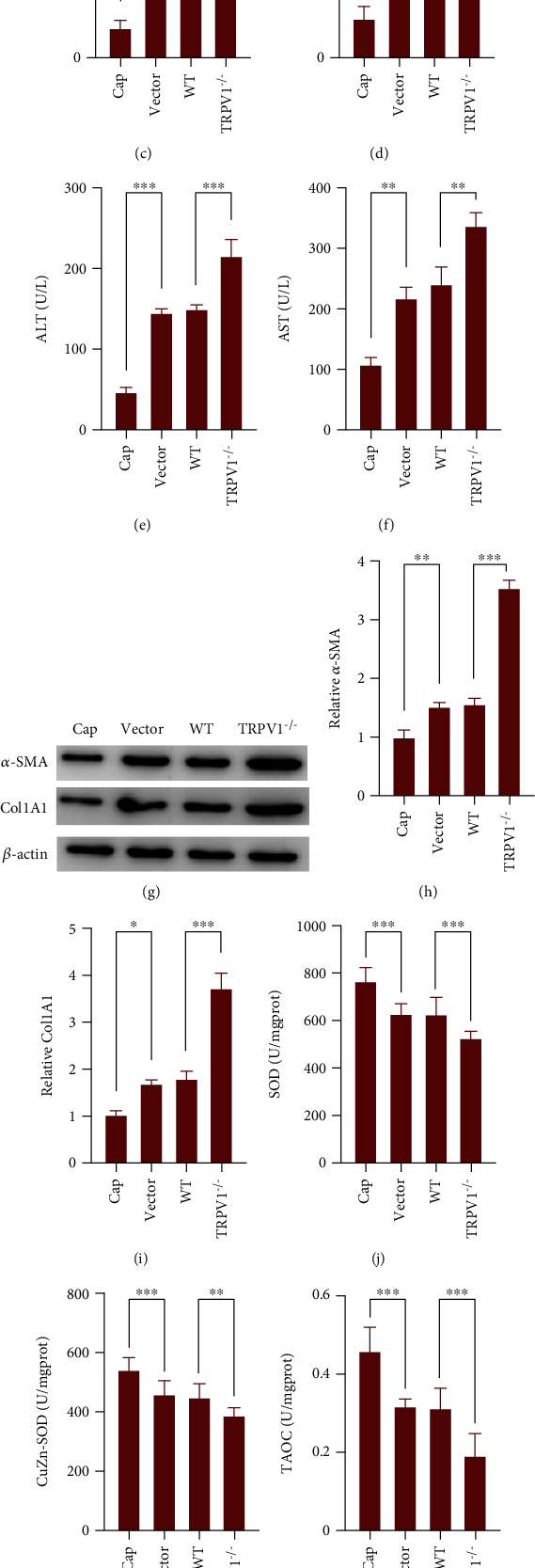
Protective effect of TRPV1 on the CCl_4_-induced mouse model. (a–d) The serum concentration levels of hyaluronic acid, collagen type IV, laminin, and precollagen type III in the indicated four groups were determined using radioimmunoassay kits. (e, f) ALT and AST in serums derived from mice from four different groups were determined by standard enzymatic assay kits. Data are presented as mean ± SD of three independent experiments. (g–i) The protein levels of *α*-SMA and COL1A1 in liver tissues from the four groups were determined using Western blot analyses. (j–n) The activity of SOD, CuZnSOD, T-AOC, GSHPX, and MDA was determined in the liver tissues of the four groups of mice. Data are presented as mean ± SD (*n* = 5 mice). ^∗^*p* < 0.05, ^∗∗^*p* < 0.01, and ^∗∗∗^*p* < 0.001.

In CCl_4_-induced fibrosis models, hyaluronic acid levels were significantly increased in *TRPV1^−/−^* mice when compared with WT, whereas the capsaicin treatment group demonstrated significantly suppressed expression when compared with its vector (*p* < 0.01) (Figure 2(a)). Similar findings were observed on other fibrosis markers, such as collagen type IV (Figure 2(b)), precollagen type III (Figure 2(c)), laminin (Figure 2(d)), and liver injury indicators, such as ALT (Figure 2(e)) and AST (Figure 2(f)). The obtained results indicated that *TRPV1^−/−^* had developed an exacerbated phenotype under CCl_4_-induced hepatic fibrosis.

Moreover, serum levels of ALT and AST were significantly upregulated in TRPV1^*−*/*−*^ mice whereas levels were downregulated in WT mice with capsaicin intraperitoneal injection, compared with WT with standard intraperitoneal injection (*p* < 0.01) (Figures 2(e) and 2(f)), suggesting that TRPV1 KO further exacerbated CCl_4_-induced liver injury. To further confirm the effect of TRPV1 on CCl_4_-induced hepatic fibrosis, we evaluated the effect of capsaicin-induced TRPV1 activation and TRPV1 KO on CCl_4_-induced hepatic fibrosis by measuring the protein expression of fibrosis markers, including *α*-SMA and COL1A1. The results showed that capsaicin intake reduced CCl_4_-induced *α*-SMA and COL1A1 upregulation in WT mice; however, in *TRPV1^−/−^* mice, CCl_4_-induced *α*-SMA and COL1A1 upregulation were further enhanced (*p* < 0.05) (Figures 2(g)–2(i)). The obtained results suggest that capsaicin may improve CCl_4_-induced hepatic fibrosis through activating TRPV1, whereas TRPV1 KO could enhance CCl_4_-induced hepatic fibrosis.

### 3.4. Effects of TRPV1 Activation and Knockout on the Antioxidant Metabolism of the CCl_4_-Induced Hepatic Fibrosis Mouse Model

As illustrated in Figures 2(j)–2(m), the obtained results suggest that comparing with the control group, SOD, CuZnSOD, T-AOC, and GSHPX in the capsaicin-induced TRPV1 group were significantly increased, while these essential markers of antioxidant metabolism in the TRPV1 KO group were significantly reduced (*p* < 0.01). However, an opposite trend was observed for MDA, that is, suppression was found in the capsaicin-induced TRPV1 group while significant elevation in *TRPV1^−/−^* groups (*p* < 0.01), as shown in Figure 2(n).

### 3.5. The Expression of TRPV1 in TGF-*β*-Stimulated HSCs and TRPV1 Knockdown Enhances TGF-*β*1-Induced ECM Protein Expression

**Figure 3:**
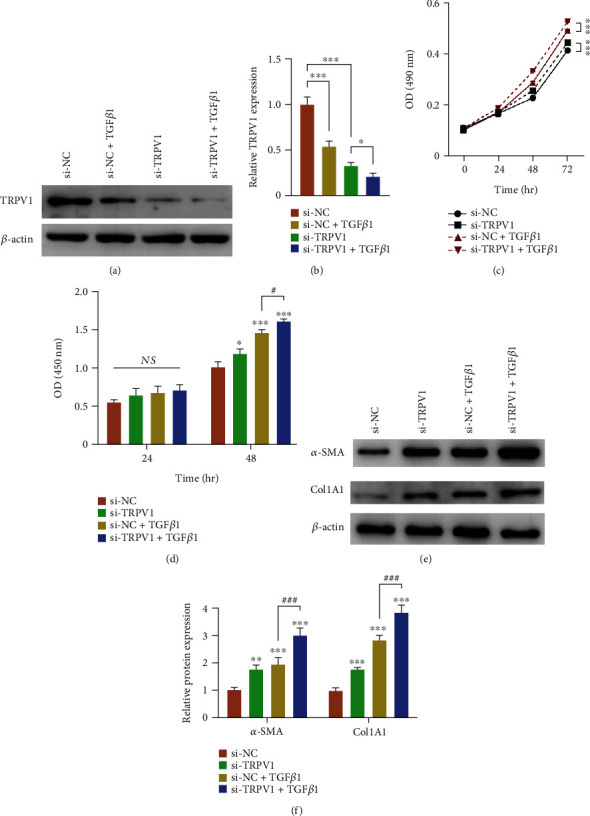
The expression of TRPV1 in TGF-*β*-treated HSCs and TRPV1 knockdown enhances TGF-*β*-induced ECM protein expression. (a, b) HSCs were exposed to 5 ng/ml TGF-*β* stimulation or (and) transfected with si-TRPV1; TRPV1 protein expression in HSCs was determined through Western blot analysis. Data are presented as mean ± SD (*n* = 5, ^∗^*p* < 0.05, ^∗∗∗^*p* < 0.001). (c) The proliferation of si-TRPV1-transfected HSC in the presence or absence of TGF-*β* stimulation was determined using MTT assay. The obtained results are presented as mean ± SD (*n* = 5, ^∗∗∗^*p* < 0.001). (d) The proliferation of HSC was determined using BrdU assay. The obtained results are presented as mean ± SD (*n* = 5, ^∗^*p* < 0.05, ^∗∗∗^*p* < 0.001, compared with the si-NC (negative control) group; ^#^*p* < 0.05, compared with the si-NC+TGF-*β* group). (e, f) The protein levels of *α*-SMA and COL1A1 in si-TRPV1-transfected HSC in the presence or absence of TGF-*β*1 stimulation were determined using Western blots. Data are presented as mean ± SD (*n* = 5, ^∗∗^*p* < 0.01, ^∗∗∗^*p* < 0.001 compared with the si-NC group; ^###^*p* < 0.001, compared with the si-NC+TGF-*β* group).

After confirming that TRPV1 KO exacerbated CCl_4_-induced liver injury and hepatic fibrosis, its expression and function in TGF-*β*-stimulated HSCs were measured. As shown in Figures 3(a) and 3(b), the TRPV1 level was significantly decreased in TGF-*β*-treated HSCs (*p* < 0.05). HSCs were then transfected with si-TRPV1 to achieve TRPV1 knockdown. Herein, MTT and BrdU assays were performed to assess the combined effect of TGF-*β* treatment and TRPV1 knockdown on HSC proliferation. As shown in Figures 3(c) and 3(d), HSC proliferation was significantly promoted by either TRPV1 knockdown or TGF-*β* treatment (*p* < 0.05), whereas an even stronger promotion effect was noted when coprocessing si-TRPV1 transfection and TGF-*β* treatment (*p* < 0.05), which suggests that TRPV1 knockdown may enhance the promotive effect of TGF-*β* treatment on HSC proliferation. To study the association between TRPV1 knockdown in ECM protein expression under the influence of TGF-*β*, HSCs were transfected with si-TRPV1 in the presence or absence of TGF-*β* treatment and examined for the protein levels of TRPV1, *α*-SMA, and COL1A1. Consistent with previous studies, either TRPV1 knockdown or TGF-*β* significantly promoted ECM protein levels, including *α*-SMA and COL1A1, whereas TRPV1 knockdown even enhanced the promotive effect of TGF-*β* on *α*-SMA and COL1A1 expressions (*p* < 0.01) (Figures 3(e) and 3(f)). Data suggested that TRPV1 knockdown could further promote *α*-SMA and COL1A1 protein expression upon TGF-*β* stimulation.

### 3.6. Capsaicin Can Partially Reverse the Effect of TGF-*β* on HSCs

**Figure 4:**
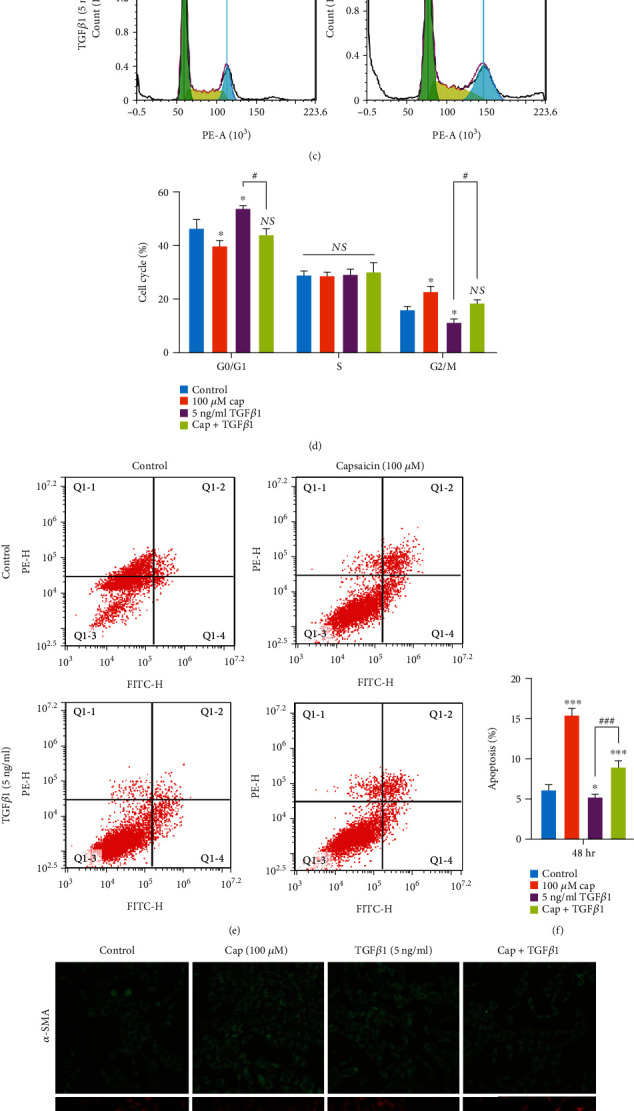
Capsaicin can partially reverse the effect of TGF-*β* on HSCs. (a, b) HSCs were cotreated with 5 ng/ml TGF-*β* and a series of doses of capsaicin (0, 50, and 100 *μ*M); HSC proliferation was determined using MTT and BrdU assays. (c, d) HSCs were treated with 100 *μ*M capsaicin or 5 ng/ml TGF-*β* or cotreated by both; the cell cycle of HSCs was determined using flow cytometry. (e, f) The cell apoptosis rate was determined using flow cytometry. (g) *α*-SMA and COL1A1 contents were shown using IF assays. (h, i) Western blot analysis on protein levels of *α*-SMA and COL1A1 was conducted. Data are presented as mean ± SD of five independently repeated experiments. ^∗^*p* < 0.1, ^∗∗∗^*p* < 0.001, compared with the control group; ^#^*p* < 0.1, ^###^*p* < 0.001, compared with the 0 *μ*M capsaicin+TGF-*β* group or TGF-*β* only group.

To investigate if capsaicin is capable of reversing the effect of TGF-*β* on HSCs, HSCs were cotreated with 5 ng/ml TGF-*β* plus a series of variable capsaicin doses of 0, 50, and 100 *μ*M. Results obtained from MTT and BrdU assays showed that capsaicin could reverse the promotive effect of TGF-*β* on HSC proliferation in a dose-dependent manner (Figures 4(a) and 4(b)), suggesting that TRPV1 activation may alleviate the effect of TGF-*β* on HSC proliferation.

Furthermore, HSCs were treated with 100 *μ*M capsaicin or 5 ng/ml TGF-*β* or cotreated by both; the cell cycle and cell apoptosis were monitored. Capsaicin treatment induced cell cycle arrest at the G2 phase, while TGF-*β* could remove the inducible effect of capsaicin (Figures 4(c) and 4(d)). As revealed by flow cytometry, TGF-*β* remarkably reduced the cell apoptosis rate while capsaicin significantly promoted cell apoptosis of HSCs; the effect of TGF-*β* could be partially reversed by capsaicin treatment (Figures 4(e) and 4(f)). The obtained results suggest that the effect of TGF-*β* on HSC proliferation, cell cycle, and cell apoptosis could be partially reversed by activating TRPV1 by capsaicin. Moreover, IF assays also revealed that once HSCs were exposed to TGF-*β* stimulation, cells quickly started to exhibit a spindled-shaped morphology and such morphological shape change process was reversible after the introduction of capsaicin. The fluorescence intensity representing *α*-SMA and COL1A1 in TGF-*β*-stimulated HSCs was enhanced compared with that of the control group, whereas it was weakened by capsaicin treatment; the effect of TGF-*β* stimulation on the HSC shape and fluorescence intensity representing ECM proteins could be partially reversed by capsaicin treatment (Figure 4(g)). To verify the fluorescent observations, Western blot analyses were also conducted and the obtained results have further corroborated that both *α*-SMA and COL1A1 levels were first significantly elevated due to TGF-*β* stimulation (*p* < 0.001) and started to decrease after capsaicin was introduced (*p* < 0.001) as shown in Figures 4(h) and 4(i). Collectively, our findings suggest that the promotive effects of TGF-*β* stimulation on *α*-SMA and COL1A1 can be partially reversed by an exogenous TRPV1 activator, i.e., capsaicin.

## 4. Discussion

In the present study, we demonstrated the detailed function of TRPV1 in the CCl_4_-induced hepatic fibrosis model of mice and TGF-*β*-induced HSCs by measurement of liver injury markers, hepatic fibrosis markers, and HSC proliferation. So far, no animal model can fully simulate human liver disease. [39] Nevertheless, animal models have been widely used in research because of several advantages, such as the convenience of collecting specimens, controlling variables as much as possible, and studying a particular gene. The CCl_4_-induced model of liver fibrosis is a widely used and studied, reliable animal model of hepatic fibrosis [2, 12, 13]. After knocking out TRPV1, CCl_4_-induced liver injury and hepatic fibrosis aggravated and TGF-*β*-induced *α*-SMA and COL1A1 protein expression and HSC proliferation were significantly enhanced. On the contrary, one of the TRPV1 agonists, capsaicin, can partially reverse the effect of CCl_4_ on the aforementioned indicators, further implying that capsaicin may activate TRPV1, which may affect the function of CCl_4_ in hepatic fibrosis and TGF-*β* in HSC activation.

We have also established a CCl_4_-induced hepatic fibrosis model using WT or *TRPV1^−/−^* mice to evaluate the functional role of TRPV1 in hepatic fibrosis. After CCl_4_ injection, both WT with standard intraperitoneal injection and *TRPV1^−/−^* mice acquired typical hepatic fibrosis morphology. However, *TRPV1^−/−^* mice had more severe hepatic fibrosis whereas capsaicin intraperitoneal injection ameliorated CCl_4_-induced hepatic fibrosis. Moreover, *TRPV1^−/−^* also aggravated CCl_4_-induced upregulation of serum hyaluronic acid, collagen type IV, laminin, precollagen type III, ALT, and AST; all of which are generally considered indicators of hepatic function and hepatic fibrosis [40], implying that TRPV1 might protect against CCl_4_-induced hepatic fibrosis.

Importantly, the serum levels of the aforementioned indicators were significantly improved by capsaicin intraperitoneal injection, also suggesting the protective role of TRPV1 against hepatic fibrosis. Herein, CCl_4_-induced changes in hepatic fibrosis or liver injury markers could all be attenuated by capsaicin intraperitoneal injection in WT mice, suggesting that capsaicin can activate TRPV1, thereby affecting the effect of CCl_4_ on hepatic fibrosis. Furthermore, by measuring the protein levels of ECM markers, *α*-SMA and COL1A1, we revealed that capsaicin can attenuate, whereas TRPV1 KO mice could enhance, the effect of CCl_4_ on hepatic fibrosis, indicating that TRPV1 plays a protective role against CCl_4_-induced hepatic fibrosis.

The paradigm of HSCs remains the most critical cell type responsible for the excessive collagen synthesis found in hepatic fibrosis [5, 41–47]. HSCs can be either transformed or activated after liver injury through a complex progression to become the myofibroblast-like phenotype. Thereafter, abundant ECM is synthesized by fibroblasts and results in hepatic fibrosis. During the progression of hepatic fibrosis, the presence of *α*-SMA, a cytoskeletal protein, has been considered a HSC activity marker [7, 41, 48, 49]. Consistent with previous studies [7, 41, 44, 50], TGF-*β* is capably of not only inducing HSC activation, which resulted in an elevated ECM expression, such as *α*-SMA and COL1A1, but also promoting the proliferation of the activated HSCs. Notably, the obtained evidence, such as enhanced ECM production and HSC proliferation, has led to the conclusion that TRPV1 knockdown may aggravate the TGF-*β*-induced HSC activation and proliferation.

Another major finding of the study is capsaicin's ability to reverse the promotive effect of TGF-*β* on ECM production, cell proliferation, cell cycle, and cell apoptosis which appears to be dose dependent. However, only partial reversal was realized. An additional experimental work is needed to address the possibility of achieving full reversal through a better understanding of the interaction between TGF-*β* activation and capsaicin attenuation.

Altogether, the obtained data strongly suggest that the correct TRPV1 modulation can improve CCl_4_-induced hepatic fibrosis and attenuate the effect of TGF-*β* on HSC activation and proliferation. In conclusion, our study suggests that TRPV1 may potentially be a promising target for developing new therapeutic strategies to treat hepatic fibrosis.

## Data Availability

The datasets used and analyzed during the current study are available from the corresponding author on reasonable request.
